# Sex differences in the rate of fatigue development and recovery

**DOI:** 10.1186/1476-5918-5-2

**Published:** 2006-01-16

**Authors:** WJ Albert, AT Wrigley, RB McLean, GG Sleivert

**Affiliations:** 1 Human Performance Laboratory, Faculty of Kinesiolgy, University of New Brunswick, Fredericton, New Brunswick, Canada; 2 PacificSport, Canadian Sport Centre, Victoria, British Columbia, Canada

## Abstract

**Background:**

Many musculoskeltal injuries in the workplace have been attributed to the repetitive loading of muscle and soft tissues. It is not disputed that muscular fatigue is a risk factor for musculoskeltal injury, however the disparity between gender with respect to muscular fatigability and rate of recovery is not well understood. Current health and safety guidelines do not account for sex differences in fatiguability and may be predisposing one gender to greater risk. The purpose of this study was to quantify the sex differences in fatigue development and recovery rate of lower and upper body musculature after repeated bouts of sustained isometric contractions.

**Methods:**

Twenty-seven healthy males (n = 12) and females (n = 15) underwent bilateral localized fatigue of either the knee extensors (male: n = 8; female: n = 8), elbow flexors (male: n = 8; female: n = 10), or both muscle groups. The fatigue protocol consisted of ten 30-second sub-maximal isometric contractions. The changes in maximum voluntary contraction (MVC), electrically evoked twitches, and motor unit activation (MUA) were assessed along with the ability to control the sustained contractions (SLP) during the fatigue protocol using a mixed four-factor repeated measures ANOVA (gender × side × muscle × time) design with significance set at p < 0.05.

**Results:**

There was a significant loss of MVC, MUA, and evoked twitch amplitude from pre- to post-fatigue in both the arms and legs. Males had greater relative loss of isometric force, a higher rate of fatigue development, and were less capable of maintaining the fatiguing contractions in the legs when compared to the females.

**Conclusion:**

The nature of the induced fatigue was a combination of central and peripheral fatigue that did not fully recover over a 45-minute period. The results appear to reflect sex differences that are peripheral, and partially support the muscle mass hypothesis for explaining differences in muscular fatigue.

## Background

Muscular fatigue is a complex process that is most often defined as an exercise induced reduction in the ability of a muscle to generate force [1,2], and has been studied over numerous exercises for decades in an attempt to understand and identify the mechanisms that lead to the loss of force production [1]. Muscle fatigue can occur centrally through the impairment of central drive and neuromuscular propagation, or peripherally through the impairment of muscle function, and more specifically excitation-contraction coupling impairment [2,3]. Therefore, distinctions must be made with respect to the nature of the observed fatigue. Central fatigue can be defined as a decline in the ability to activate the muscle during exercise whereas peripheral fatigue can be defined as the impairment of any process distal to the neuromuscular junction [2].

Muscle fatigue is most commonly assessed through the loss of force production [4] since maximum voluntary contractions (MVC) quantify the end result of both central and peripheral processes resulting in generating a muscular contraction [1]. It has also been assessed by changes in frequency measures of electromyography (EMG) signals [5,6], the reduction in electrically evocable forces [7], changes in metabolic factors [8,9], or with ratings of perceived exertion [6]. Females have been shown to have a significant advantage in relative fatigability due to reduced absolute force loss [4], but no significant differences have been found with respect to both metabolic factors [9] and ratings of perceived exertion [6]. Unrelated to fatigue, sex differences in EMG frequency measures have been difficult to determine [5].

The majority of workplace musculoskeletal injuries are related to repetitive strain [10], however, the individual effects of peripheral and central fatigue have not isolated. In manual materials handling work for example, the fatigue related changes in lifting technique has been a key focus [11-15]. due to the perceived injury risk of fatigued muscles being less capable of reacting to any perturbations that may occur during a lift [12,14]. The changes in lifting technique resulting from muscular strength and fatigability differences may also influence trunk motion and spinal loading [16], although it has been difficult to make this link empirically [6,17,18].

Although recent studies have shown that women have greater muscular endurance [9,19], there is a lack of information on sex differences in fatigue patterns considering the amount of information documenting strength differences [4]. The mechanisms for these differences are largely unknown, but there are two widely proposed hypotheses; 1) differences in muscle mass, and 2) differences in activation pattern [19]. Recent research [9,19] appears to indicate that the first hypothesis is most likely related to the sex differences in muscular fatigue due to similar findings that differences in endurance times during a fatiguing task were not related to differences in the neuromuscular recruitment strategy when both men and women were assessed. Therefore, it is most likely that the increased absolute force generated by men causes a greater demand for muscle oxygen with more occlusion of blood flow due to increased compression of tissues [19]. Current physiological guidelines for work to rest ratios and level of activity such as lifting are based on the metabolic demand of the activity [20] and do not consider sex differences in fatigue development or recovery.

The absolute levels of muscular force production and fatigability disparity between genders may be a predisposition to a greater risk of injury when required to work in a fatigued state. To address this fundamental issue a protocol for inducing muscular fatigue must be used to assess the nature of fatigue induced (peripheral versus central), the extent of the fatigue, rate of fatigue development, rate of recovery, and whether there are sex differences for all of these measures across multiple muscle groups. Therefore, the purpose of this study was to quantify the sex differences in fatigue development and recovery rate of lower and upper body musculature after repeated bouts of sustained isometric contractions. It is hypothesized that sex differences in the rates of fatigue accumulation and recovery of different muscle groups will lead to gender specific limiting neuromuscular factors for occupational tasks.

## Methods

### Participants

Twelve healthy males (age: 24.5 ± 2.5 years.; weight: 81.5 ± 9.3 kg; height: 177.6 ± 6.1 cm; Body Mass Index: 25.8 ± 2.0 kg/m^2^) and 15 healthy females (age: 23.5 ± 2.4 years.; weight: 64.0 ± 6.9 kg; height: 169.8 ± 4.8 cm; Body Mass Index: 22.3 ± 3.1 kg/m^2^) underwent bilateral localized fatigue of either the knee extensors (male: n = 8; female: n = 8), elbow flexors (male: n = 8; female: n = 10), or both muscle groups. All physical characteristics were measured in accordance with the Canadian Physical Activity Fitness and Lifestyle guidelines [21] using a wall mounted metric tape (Seca Body Meter) and a beam scale to obtain height and weight, respectively. Due to difficulties in retaining participants, only four males and three females participated in both conditions. All participants provided written informed consent in accordance with guidelines set by the university Ethics Review Board.

### Determination of maximum voluntary contraction

**Figure 1:**
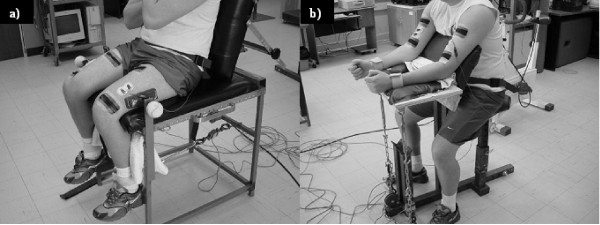
All force recordings were performed while the participants were secured to custom-built isometric knee extensor (a) and elbow flexor (b) myographs fitted with two independent force transducers. The force transducers were secured to the limbs using canvas straps and allowed for simultaneous recording of the net reaction force acting through the transducer at the site of attachment required to balance the torque generated by the muscular contractions.

All force recordings were performed while the participants were secured to custom-built isometric knee extensor and elbow flexor myographs (Figure 1) fitted with two independent PT4000-500lb force transducers (Precision Transducers, Auckland, New Zealand) that were amplified (MM50, Micron Meters, Simi Valley, CA) and analogue to digital converted through a NI-6036E Multifunction DAQ board (National Instruments, Austin, TX) housed on a Pentium II desktop PC at 1024 Hz. The force transducers were secured to the limbs using canvas straps with heavy duty Velcro, and allowed for simultaneous recording of the amplified raw net reaction force acting through the transducer at the site of attachment required to balance the torque generated by the muscular contractions. For the isometric knee extensions, the straps were placed around the distal shank such that the transducers were aligned parallel to the floor, with the knee secured at an angle of 110° of flexion from the horizontal (Figure 1a). As for the isometric elbow flexions, the straps were placed around the distal forearm such that the transducers were aligned vertically, with the elbow secured at an angle of 120° of extension from the upper arm (Figure 1b). The testing devices were placed in front of the computer and the monitor showed a real-time graphical display of the two independent forces produced by each of the limbs using custom software created with LabView 6.0 (National Instruments, Austin, TX). The bilateral knee extension and elbow flexion tests were performed seperately, in a randomized order. In each test, participants were required to complete three MVCs with both limbs simultaneously with a minimum of three minutes of rest between contractions. For the knee extensor contractions, the participants were required to fold their arms across their chest. During both the knee extensor and elbow flexor contractions, the participants were provided with verbal encouragement from at least one researcher. In order to attenuate the influence of high frequency oscillations in the force recordings introduced by the amplifier, all force signals were low pass filtered using a zero lag 4^th ^order Butterworth filter with a cut-off frequency of 25 Hz prior to extracting any information. The maximum voluntary force produced over the three contractions was used as the MVC.

### Fatigue and recovery protocol

**Figure 2:**
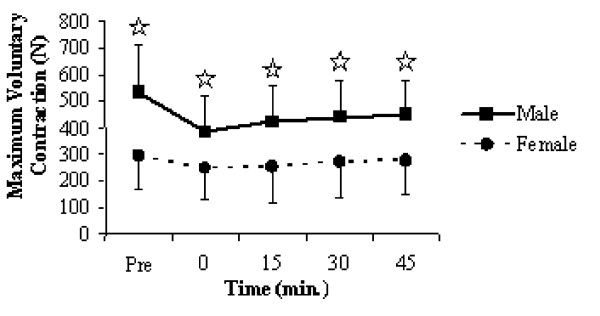
**a) **Truncated force signal trace illustrating the fatigue protocol. (♠) The peak of three 5-second maximum voluntary contractions (MVC) was used to determine the fatiguing contraction intensity, while the interpolated twitch amplitude (IT) along with the associated control twitch (CT) elicited 5-seconds after the contraction were used to calculate the pre-fatigue level of motor unit activation. (♥) Ten 30-second fatiguing contractions with 30-seconds of rest were performed at 50% of MVC with an evoked twitch (ET) elicited 5-seonds after the contraction. The slope of the force plateau (SLP) was used as an indicator of fatigue accumulation. (♦) Recovery was monitored for 45-minutes where an MVC with IT was performed every 15-minutes, with ETs elicited at 5-minute intervals. **b) **Typical trace for one second of raw EMG data illustrating the 0.25 second overlapping epochs utilized to calculate mean root mean square and mean median frequency values.

The fatigue protocol followed the completion of electrode placement for both electrical stimulation and EMG recording, and the determination of the participant's MVC. While still secured in the respective isometric myograph, the participants were instructed to perform and maintain ten 30-second isometric contractions at 50% of their previously determined MVC, with 30-seconds of rest between contractions. If the participant was unable to maintain the required force level, they were verbally encouraged to continue with the contraction until the 30-second mark. Visual feedback was provided by custom software created with LabView 6.0 (National Instruments, Austin, TX) that displayed a real-time plot of the recorded force signals, with a target line placed at 50% of the pre-determined MVC. Verbal feedback was also provided throughout the contractions in order to assist the participant with maintaining control over both limbs. During the elbow flexion task, participants were given a light wooden dowel to hold in order to ensure that the forearms remained supinated. Immediately following the tenth fatiguing contraction and every 15 minutes afterwards up to 45-minutes, participants were required to perform five-second MVCs to determine voluntary strength loss, changes in central activation, and the rate of recovery of these variables. At five-minute intervals between the MVCs during the recovery period, electrically evoked doublets were recorded as an index of fatigue and recovery [22]. A graphical representation of the protocol is presented in Figure 2a.

### Electrical Stimulation

Electrical stimulation was used to perform the twitch interpolation technique [2,23,24] for quantifying the percentage motor unit activation (MUA) during all MVCs, and for evoking control doublets. Carbon rubber electrodes (4.45 cm × 10.16 cm) were placed on both limbs and delivered two 200 μs width square wave pulses from a continuously variable constant current stimulator (Digitimer DS7A, Hertfordshire, United Kingdom) separated by 10 ms. The electrodes were coated in a conducting gel and secured to the limb with tape. For the knee extension task, the cathode was placed across the lateral proximal quadriceps femoris muscle group approximately 3 cm from the anterior superior iliac spine, with the anode placed across the medial distal aspect approximately 2 cm from the superior border of the patella [25]. Due to the size of the electrodes relative to the surface area of the biceps brachii muscle group, the cathode was placed across the midline of the muscles below the ridge created by the deltoid and the anode was placed across the midline of the muscles above the anticubital space. The electrical stimulus was recorded as a 5 V pulse and collected simultaneously with the force signals.

It has been suggested that using an absolute supramaximal stimulation of the biceps brachii for twitch interpolation may stimulate the triceps brachii which may in turn affect the calculated MUA [26]. Therefore, in order to determine the greatest amount of twitch force that could be electrically evoked in the elbow flexors, the current was increased from 200 mA in 25 mA increments until no further increase in force was observed. The intent was to reach maximal stimulation of the biceps brachii while employing a current that is adapted to each individual thereby controlling for differences in muscle thickness. A similar protocol was employed for the knee extensors where the current was increased from 250 mA in 50 mA increments. During each of the three MVCs performed prior to the fatiguing contractions and the four MVCs performed during recovery, the electrical stimulus was applied at the anticipated peak of the force and five seconds after the contraction was completed. During the 10 fatiguing contractions the electrical stimulus was applied at the 25 second mark and again five seconds after the contraction was completed. During the recovery period, doublets were evoked every five minutes, excluding those intervals when a MVC was performed.

### EMG electrode placement and measurement

Two channels of an EMG cable telemetry system (Octopus, Bortec Biomedical Ltd., Alberta, Canada) connected to the National Instruments 32 channel A/D board were used to amplify (by a factor of two thousand) and collect all raw EMG signals at 1024 Hz, synchronized with the force and electrical stimulus data. The surface EMG was collected using a bipolar configuration of silver/silver chloride electrodes (Red Dot, 3 M Canada, London, ON) placed on the vastus lateralis during the knee extension task and on the short head of the biceps brachii during the elbow flexion task. For the knee extension task, the muscle belly of the vastus lateralis was palpated, and point stimulation was utilized to locate the innervation zone. A single 200 μs wide square wave pulse was applied at 40 mA using a constant current stimulator (Digitimer DS7A, Hertfordshire, United Kingdom) along the muscle belly and the site of the greatest muscular twitch was marked, denoting the innervation zone. The EMG electrodes were then placed on the middle of the muscle belly parallel with muscle fibres between the innervation zone and musculotendonous junction at an inter-electrode distance of 2 cm [27]. A single reference electrode was placed along the medial aspect of the right tibia. However, due to the limited surface area available on the biceps brachii muscle group, the EMG electrodes were placed along the surface of the short head between the two carbon rubber stimulating electrodes, with the reference electrode placed on the right acromion. All sites of EMG electrode placement were shaved, swabbed with alcohol and abraded to minimize potential electrical resistance [28].

The raw EMG data from the fatiguing contractions was processed using a zero lag 4^th ^order band pass Butterworth filter with low and high cut-off frequencies set to those recommended by Basmaijian and De Luca [28] for surface electrodes of 20 Hz and 500 Hz respectively. In order to determine the mean root mean square (MRMS_30_) and mean median frequency (MMF_30_) of each fatiguing contraction, the 30-second signal was broken into overlapping epochs of 0.25 seconds (Figure 2b) [5]. A mean root mean square (MRMS_1_) was then determined for each one-second increment by calculating the average of the seven root mean square values in each of the appropriate epochs. Finally, the MRMS_30 _was calculated by taking the average of the 30 MRMS_1 _values. Similarly, after the time signal was transformed into the frequency domain through a fast Fourier transform (512 point Hamming window), the MMF_30 _of each fatiguing contraction was calculated using the same process. All signal processing and calculations were performed using custom software created in LabView 6.0 (National Instruments, Austin, TX).

### Statistical analysis

The dependent measures analyzed for both tasks were maximum voluntary contraction (MVC), percent MVC, motor unit activation (MUA), and electrically evoked twitches from pre-fatigue to post-fatigue (Figure 2a). The electrically evoked twitches were also analyzed throughout the 10 fatiguing contractions. Furthermore, the ability to maintain 50% of MVC during the fatiguing contractions (SLP) was assessed by calculating the slope of the force signal after the rising and falling edges were removed (Figure 2a), and the lines of best fit from a linear regression for the MRMS_30 _and MMF_30 _over the 10 fatiguing contractions (MRMS_slope _and MMF_slope _respectively) were analyzed. The MVC, percent MVC, MUA, twitches, and SLP variables were analyzed using mixed four-factor repeated measures ANOVA (gender × side × muscle × time) designs (it should be noted that only the time × muscle and sex × muscle interactions were of interest with respect to percent MVC). Due to the small sample sizes and their effect on Mauchly's test of sphericity, the Greenhouse-Geisser epsilon was calculated and utilized to adjust the degrees of freedom in order to avoid the assumptions made about the variance-covariance matrices of the dependent variables (SPSS for Windows v11.5, ^©^SPSS Inc. 1989–2002). Therefore, significance of the repeated-measures tests were derived using the adjusted degrees of freedom. Any within- or between-subjects main effects were assessed using pairwise comparisons with Bonferonni corrections for multiple comparisons, and time interactions with sex, side, and muscle were assessed using independent samples T-tests. The MRMS_slope _and MMF_slope _were analyzed using a one-sample T-test for group differences. Significance was accepted at *P *< 0.05.

## Results

Descriptive data (mean and standard deviation) for the pre/post measures of MVC, MUA, and evoked twitches are presented in Table 1, whereas results for the evoked twitches and SLP derived for the fatiguing contractions are presented in Table 2.

**Table 1:** Descriptive measures from pre- to post-fatigue for maximum voluntary contraction (MVC), motor unit activation (MUA), and doublet twitch amplitude. Values are mean (standard deviation) for both genders and muscle groups. Time is measured in minutes, and corresponds to the duration after the tenth fatiguing contraction was performed that the measure was taken.

		Elbow Flexors		Knee Extensors	
		
Variable	Time	Female	Male	Female	Male
MVC (N)	Pre*	197.4(31.5)	364.6(64.9)	438.9(70.1)	677.4(102.4)
	0*	158.6(38.4)	262.2(60.8)	379.3(60.9)	487.9(86.2)
	15*	154.6(59.2)	311.9(80.3)	392.9(85.7)	517.7(90.2)
	30*	174.3(36.9)	317.7(81.3)	406.9(79.7)	543.4(81.5)
	45*	185.6(36.9)	324.6(55.8)	406.4(92.3)	553.7(70.8)
					
MUA (%)	Pre	93.6(3.3)	94.9(3.9)	86.2(10.8)	91.3(6.6)
	0	85.1(11.8)	86.6(7.8)	89.8(4.7)	84.3(6.7)
	15	84.2(10.5)	91.4(5.9)	89.6(6.5)	86.1(10.6)
	30	90.4(5.9)	91.5(7.7)	89.8(6.1)	85.8(9.8)
	45	91.3(3.4)	91.5(4.5)	88.4(8.6)	87.8(8.7)
					
Twitch (N)	Pre*	25.3(9.8)	70.4(22.0)	207.3(32.9)	310.0(73.4)
	0	21.3(18.6)	34.3(21.8)	140.9(27.9)	132.4(37.3)
	15	13.8(11.4)	41.2(21.9)	135.9(24.8)	158.8(70.6)
	30*	17.0(10.9)	40.1(21.3)	150.6(26.6)	202.5(36.8)
	45*	18.0(8.9)	38.1(20.5)	154.9(24.8)	205.2(30.7)

* Indicates significant difference (*P *< 0.05) between genders when muscles are grouped.

**Table 2:** Descriptive measures during fatigue development for evoked twitches and the ability to maintain the required force level during the contractions (SLP). Values are mean(standard deviation) for both genders and muscle groups.

		Elbow Flexors		Knee Extensors	
		
Variable	Contraction	Female	Male	Female	Male
Twitch (N)	Pre*	25.3(9.8)	70.4(22.0)	207.3(32.9)	310.0(73.4)
	1*	22.9(10.7)	59.2(17.2)	180.0(29.9)	252.5(31.5)
	2*	21.9(11.0)	50.3(15.7)	172.2(28.2)	223.5(35.7)
	3*	23.4(8.5)	48.1(16.7)	150.6(50.8)	207.6(43.7)
	4	22.5(10.9)	41.2(11.3)	166.0(25.6)	185.7(39.9)
	5	21.8(7.7)	40.7(12.3)	157.7(32.4)	170.1(42.2)
	6	23.0(9.4)	35.7(14.1)	148.8(24.3)	150.5(39.9)
	7	20.6(12.0)	31.3(13.0)	146.0(28.0)	143.2(41.1)
	8	18.3(6.0)	31.2(13.1)	122.2(49.9)	130.2(44.8)
	9	18.2(10.2)	28.5(14.2)	128.8(29.7)	118.9(32.2)
	10	17.5(9.8)	25.4(14.8)	121.8(30.6)	112.0(35.9)
					
SLP (N·sec^-1^)	1	0.78(4.5)†	-0.04(0.9)	0.11(0.6)	0.36(1.0)
	2	-0.15(0.5)	-0.06(0.5)	-0.66(0.5)	0.39(2.4)
	3	-0.22(0.3)	0.01(0.6)	-0.35(0.8)	0.02(1.1)
	4	-0.44(0.5)	-0.27(0.7)	-0.21(0.8)	-0.70(0.9)
	5	-0.40(0.5)	-0.26(0.9)	0.001(1.0)	-0.45(2.2)
	6*	-0.53(0.6)	-0.77(1.6)	-0.17(0.4)	-1.68(1.67)
	7*	-0.25(0.4)	-1.08(1.9)	-0.29(0.6)	-2.16(2.22)
	8*	-0.42(0.6)	-0.15(2.8)	-0.43(0.6)	-3.30(2.18)
	9*	-0.45(0.5)	-1.05(1.6)	-0.39(0.6)	-3.32(2.9)
	10*	-0.52(0.4)	-0.89(1.5)	-0.15(0.8)	-5.12(3.3)

* Indicates significant difference (*P *< 0.05) between genders when muscles are grouped.

† A single participant initially demonstrated difficulty with the coordination of the task resulting in her SLP value for the first contraction being highly positive. Removal of this participant's data point did not influence the statistical outcome and has therefore remained in the analysis.

### Pre/Post measures

There were no significant sex differences for the arms or legs with respect to MRMS_slope_, MMF_slope_, and no gender main effect for MUA. The gender main effects for MVC (*F *= 80.696, *P *= 0.001, η_p_^2 ^= 0.590) and evoked twitches (*F *= 26.886, *P *= 0.001, η_p_^2 ^= 0.374) from pre- to post-fatigue revealed that males achieved higher force levels (both voluntary and electrically evoked) during both fatigue protocols.

**Figure 3:**
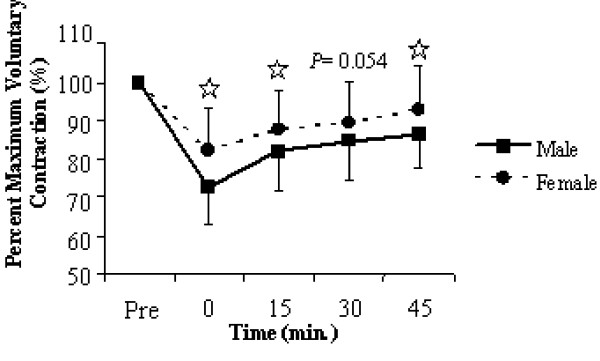
Significant time × sex interaction plot for maximum voluntary contraction from pre- to post-fatigue and throughout the 45-minutes of recovery. Time is measured in minutes and relative to the completion of the ten fatiguing contractions. Values are means ± standard deviations. Significant group differences are marked with a^☆^.

**Figure 4:**
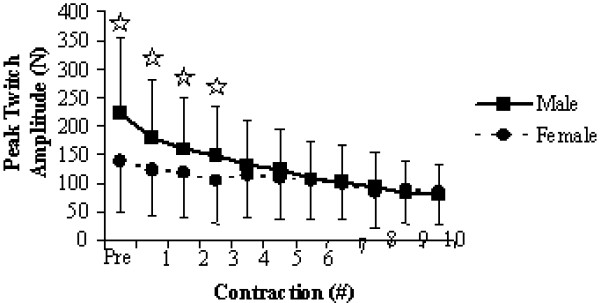
Significant time × sex interaction plot for percent maximum voluntary contraction throughout the 45-minutes of recovery. Time is measured in minutes and relative to the completion of the ten fatiguing contractions. Values are means ± standard deviations. Significant group differences are marked with a^☆^.

The significant time × gender interaction for MVC (*F *= 21.144, *P *= 0.001, η_p_^2 ^= 0.274) indicated that males achieved higher force levels at all time intervals from pre- to post-fatigue (Figure 3), yet the same interaction for evoked twitches (*F *= 19.213, *P *= 0.001, η_p_^2 ^= 0.299) revealed that the peak twitch amplitude was only greater for males at the pre-fatigue, and both the 30- and 45-minute post-fatigue intervals. The significant time × gender interaction for percent MVC (*F *= 5.228, *P *= 0.0 01, η_p_^2 ^= 0.086) revealed that females had a relative fatigue advantage (Figure 4) throughout all points of recovery except for the 30 minute interval. The gender × muscle interaction for the same variable (*F *= 5. 928, *P *= 0.018, η_p_^2 ^= 0.099) highlighted that this difference was largely attributed to difference in percent MVC for the knee extensions.

### Fatigue development

**Figure 5:**
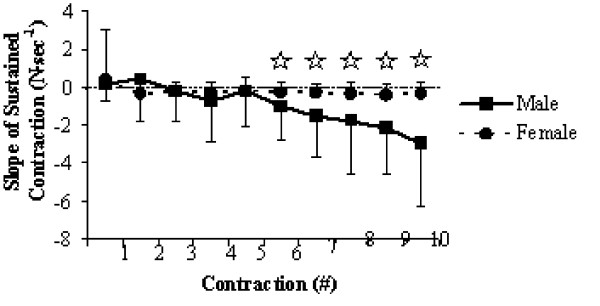
Significant time × sex interaction plot for peak evoked twitch amplitude throughout the fatiguing contractions. Values are means ± standard deviations. Significant group differences are marked with a^☆^.

The significant gender main effects during the fatiguing contractions for evoked twitches (*F *= 8.424, *P *= 0.006, η_p_^2 ^= 0.170) and SLP (*F *= 11.547, *P *= 0.001, η_p_^2 ^= 0.204) indicated that males achieved greater levels of electrically evoked force, but were less capable of maintaining the required force levels. Interestingly, the time × gender interaction for the evoked twitches (*F *= 19.516, *P *= 0.001, η_p_^2 ^= 0.322) illustrated that when compared to their male counterparts, the females had statistically equivalent peak twitch amplitudes after the third sustained contraction (Figure 5).

**Figure 6:**
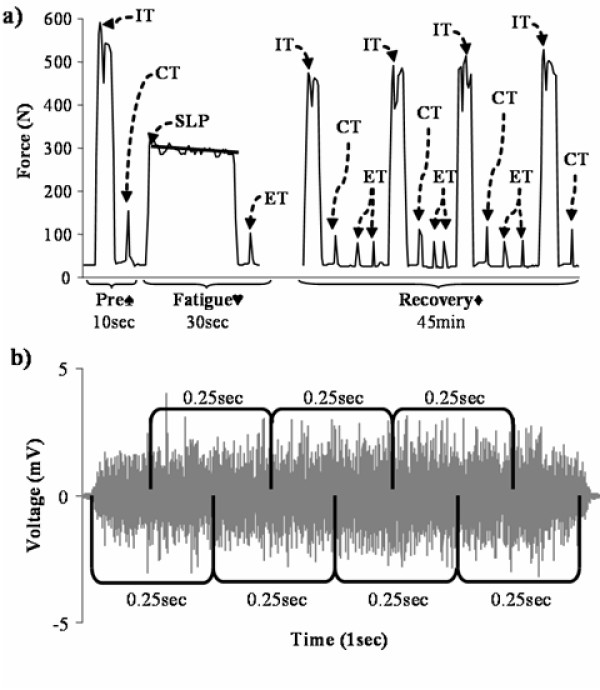
Significant time × sex interaction plot for the ability to maintain the required force level during the fatiguing contractions. Values are means ± standard deviations. Significant group differences are marked with a^☆^.

As for the ability to maintain the target force level during the fatiguing contractions, the significant time × gender interaction for SLP (*F *= 6.964, *P *= 0.001, η_p_^2 ^= 0.134) showed that the females had a significantly greater ability to maintain the required force from the sixth to tenth contractions (Figure 6). When the significant gender × muscle interaction for SLP was assessed (*F *= 5.611, *P *= 0.022, η_p_^2 ^= 0.111), it became apparent that the males had a more difficult time maintaining the target force during the isometric knee extensions as compared to the isometric elbow flexions, whereas there was no such significant difference for the females. Time × gender × muscle analysis (*F *= 5.007, *P *= 0.001, η_p_^2 ^= 0.100) revealed that the previous result was due to the inability of males to maintain the required force during the last three isometric knee extension contractions.

## Discussion

The fatigue protocol employed resulted in significant force deficits in both the elbow flexors and knee extensors to a point that they were unable to fully recover after 45-minutes of passive recovery. The absolute isometric force level achieved by males was significantly greater than that reached by females throughout the entire protocol (Figure 3). In order to compare to previously published literature, the post-fatigue MVCs were analyzed as a percentage of the pre-fatigue maximum. It became apparent that the relative isometric force for the females was significantly higher at all time intervals except at 30-minutes post-fatigue (Figure 4). Further analysis of the normalized MVC data through a gender × muscle interaction revealed that the relative fatigue advantage found for the females was only evident in the knee extensors, and not the elbow flexors. Hunter et al. [29] found that when men and women were matched for strength, similar levels of muscle fatigue and cardiovascular adjustments were observed when men and women performed submaximal isometric contractions of the elbow flexors, while Clarke [4] was able to determine that with fatiguing hand-grip exercises, males achieved higher absolute force levels with a greater force deficit and relative fatigue disadvantage when compared to females, although both groups followed a similar pattern of force decay.

Significant time × gender interactions for relative MVC from pre- to post-fatigue (Figure 3), as well as for the evoked twitches (Figure 5) and SLP (Figure 6) during the fatiguing contractions are perhaps the most functionally relevant results. The difference in relative force loss is a direct result of fatigue developing faster in the knee extensors for males, indicated by the progressive decline in evoked twitches and the inability to maintain 50% MVC for 30-seconds during the fatiguing contractions (Table 2). Previous research [30] has shown that under normal blood flow conditions, women fatigue less when performing intermittent, maximal volitional contractions with the dorsiflexor muscles. However, the measured fatigue was similar for men and women when blood flow to the muscle was occluded. Similarly, Wright et al. [31] found that when elevating the hand reduced muscle perfusion pressure; the rate of muscle fatigue was increased but was quickly reversible when perfusion pressure was restored. These results appear to reflect the muscle mass hypothesis for explaining both muscle group and sex differences in the fatigability of working muscles. The muscle mass hypothesis stems from a cascading assortment of issues that arise from males having greater muscle mass. Greater muscle mass leads to greater absolute force production, which requires more oxygen but also impairs oxygen delivery through occlusion due to increased intramuscular pressure [19]. This occlusion not only limits oxygen delivery, but also leads to the accumulation of metabolites within the working muscles [9], which all leads to an increased rate of fatigue.

Although intramuscular pressure and blood flow was not measured in the present study, some inferences could be made based on results presented in Figure 3. The significantly higher levels of voluntary force produced by the males from pre- to post-fatigue would presumably lead to higher intramuscular pressure and greater occlusion of blood flow. Interestingly, during the 45-minutes of passive recovery, the relative MVC for males and females had a very similar trend of recuperation. Therefore, the significant time × gender interaction for relative MVC from pre- to post-fatigue (Figure 4) may very well reflect similar sex differences that were identified by Russ and Kent-Braun [30]. Furthermore, the absolute force levels achieved with the knee extensors were nearly double that of the elbow flexors which would lead to a greater occlusion of blood flow for the male participants under this condition.

Due to the lack of significant sex differences found for the EMG measures that quantified fatigue development, the sex differences in the fatigability of the quadriceps muscle group could not be attributed to differences in the activation patterns of the muscle fibers. Clark et al. [19] reported similar results where sex differences in endurance time during isometric and isotonic back extensions were not related to the neuromuscular activation strategy. Furthermore, there were no sex differences for MUA from pre- to post-fatigue in the quadriceps muscle group. It should be noted that when the female knee extensor MUA data in Table 1 is plotted over time, it becomes apparent that the female subjects were not providing maximal efforts during the resting condition. Although this could have influenced the reported sex differences for the change in knee extensor MVC from pre- to post-fatigue, a lack of any statistically significant time × gender × muscle or even gender × muscle interactions for this variable suggests otherwise. If fatigue related sex differences exist for MVCs but not for MUA or EMG measures that commonly quantify fatigue development, then through a process of elimination it can be stated that differences in relative force loss can be attributed to the contraction intensity and factors distal to the activation of muscle [9]. However, as previously stated, this study did not measure all variables that could contribute to fatigue development like intramuscular pressure, blood flow, and even muscle fibre type. Therefore, it is important to note that some other mitigating factors might be present.

The technique utilized to process the EMG signals was similar to that outlined by Pincivero et al. [5], where overlapping epochs of data were analyzed to derive 'better' representations of spectral parameters. However, the present study departed somewhat in that the MMF_1 _values were averaged over each 30-second contraction to derive the MMF_30 _measure. This technique was applied because it was found that although the power spectrum of the EMG signals did shift to lower values during the fatiguing contractions, there was sufficient amount of time between contractions for the median frequency to revert back to higher values prior to commencing the subsequent contraction. This finding was not surprising as previous research determined that the electrical manifestations of fatigue recover quickly after the completion of a contraction [6]. The MMF_slope _from the vastus lateralis (right: -0.48 ± 0.6; left: -0.47 ± 0.6) were almost twice those reported by Bonato et al. [6] for the right side (-0.28 ± 0.5), without any side differences. The much larger negative slopes reported in the present study are most likely due to differences in the contraction protocol employed (ten 30-second isometric contractions at 50% MVC versus one 30-second isometric lift at 80% maximum lifting capacity) and that only males were tested in the previous study. Interestingly, the MMF_slope _from the short head of the biceps brachii were greater than those found for the vastus lateralis (right: -0.56 ± 1.5; left: -0.76 ± 1.7), but not significantly different than zero. This finding is a result of the large amount of variability found within the MMF_slope _values, as indicated by the relatively large standard deviations reported for the slopes. Although Pincivero et al. [5] found that the MF for the vastus lateralis was the most variable of the knee extensors; research was not found that reported the variability of the power spectrum for the biceps brachii muscle group. The result that the MMF_slope _was significantly negative while the MRMS_slope _was significantly positive for the vastus lateralis, but not the short head of the biceps brachii appears to indicate that there was an increase in motor unit synchronization during the fatiguing knee extensions but not in the elbow flexions [19]. Although EMG quantifies the outcome of processes originating in the central nervous system down to the excitation of muscle, but does not distinguish between central and peripheral factors that contribute to the reduced capacity to generate force [1,2], the results for the derived EMG measures appear to support those found for the rate of fatigue development.

The sex and body region muscular fatigue differences reported have physiological relevance and application in health and safety guideline developments. Current work to rest guidelines, for example, are based on the duration and frequency of an activity, but are not sensitive to the differences in the fatiguing and recovery patterns of individual body regions nor are they gender specific. It is clear from these findings that the sex differences in muscle recovery period are not well served by a single general population derived guideline and more research is needed to develop appropriate work to rest guidelines, for example, that are gender and body region specific.

## Conclusion

The major findings of this study may be summarized as follows:

• females were more fatigue resistant as indicated by the minimal change in evoked twitches during the fatiguing contractions

• females demonstrated an enhanced ability to maintain the required force levels

• both genders demonstrated significant reduction in MVC from pre- to post-fatigue and throughout the recovery period

• results appear to reflect sex differences that are peripheral to the activation of muscle
